# Telephone-based aftercare groups for family carers of people with dementia – results of the effect evaluation of a randomised controlled trial

**DOI:** 10.1186/s12913-022-07490-9

**Published:** 2022-02-11

**Authors:** Martin Berwig, Susanne Lessing, Ruth Deck

**Affiliations:** 1 Clinic for Cognitive Neurology, Medical Faculty, University of Leipzig, Liebigstr 16, 04103 Leipzig, Germany; 2 German Centre for Neurodegenerative Diseases (DZNE) – Site Witten, Witten, Germany; 3 School of Nursing Science, Faculty of Health, Witten/Herdecke University, Witten, Germany; 4 Institute of General Medicine, Otto-von-Guericke University Magdeburg, Magdeburg, Germany; 5 Institute for Social Medicine and Epidemiology, University of Lübeck, Ratzeburger Allee 160 (Haus V50), 23538 Lübeck, Germany

**Keywords:** Family carers of people with dementia, Telephone-based aftercare group sessions, Randomized controlled longitudinal study

## Abstract

**Background:**

The care of people with dementia is associated with enormous stress and, in a quarter of cases, leads to depression and anxiety disorders in the caring relatives. A specially designed inpatient psychosomatic rehabilitation (rehab) programme for family carers of people with dementia has proven to be effective but not sustainable. Therefore, the present study aims to increase the sustainability of the inpatient rehab programme by using thematically structured telephone aftercare group sessions.

**Methods:**

The effectiveness of telephone aftercare groups was investigated in a randomized, controlled, prospective, mixed methods, longitudinal study. The aftercare intervention included social participation in monthly telephone group sessions for 6 months. The primary outcome was increased social participation of family carers, which, like the secondary outcomes (such as quality of life and subjective health), was assessed in written surveys at three or four measurement points.

**Results:**

Complete data from 69 participants from the intervention group and from 72 participants from the control group could be evaluated. A small-sized reduction in restrictions on social participation was observed in the intervention group, whereas the reduction in the control group was negligible. The repeated-measures analysis of variance (ANOVA) showed sustained effects on the secondary outcomes, such as depression, perceived social support, and the mental health domain of quality of life of family carers, in favour of the intervention group. The results also showed that telephone-based aftercare groups had a rather minor influence on the use of support services. Except for those from family, friends and neighbours, existing support offers were hardly used.

**Conclusion:**

Telephone aftercare group sessions for carers of people with dementia were not able to increase social participation at the expected magnitude. Nevertheless, the clear effects on selected secondary health-related outcomes and the assessment of the telephone-based group sessions by the participants show that the caring relatives were able to benefit greatly from this aftercare measure. Family carers should be informed more extensively about the corresponding resources and encouraged to use them. Overall, this new aftercare concept can be recommended for implementation, and its use also seems to be target-oriented for other indications.

**Clinical trial registration:**

German Clinical Trials Register: DRKS00013736, 14/05/2018.

**Supplementary Information:**

The online version contains supplementary material available at 10.1186/s12913-022-07490-9.

## Background

More than two-thirds (76% or 2.59 million) of those in need of care as defined by the German social security statute book XI (Sozialgesetzbuch XI, SGB XI) in 2017 were cared for at home [1]. No specific data on people in need of care with dementia are available from the German Federal Statistical Office. It is often stated that two-thirds of dementia patients are cared for by family carers at home [2] and that family carers thus make the largest contribution to the care of this patient group in Germany, but no current studies are available on this topic.

A recent study by Karg and colleagues [3] corroborated findings from previous research [4, 5], suggesting poorer health-related outcomes such as greater subjective burden, higher levels of depressiveness and lower care-related quality of life levels among family carers of people with dementia than among family carers of people with another chronic disease. The general burden of family carers has been shown to be determined by the severity of the patient’s neuropsychiatric symptoms and the carer’s sense of competence and health-related quality of life [6].

Based on psychological models of carer stress, a variety of psychological interventions have been developed with the goal of helping family carers of relatives with dementia with their difficult tasks [7]. These interventions differ in terms of characteristics such as their format (individual or group) as well as the content (psychoeducation, counselling and psychotherapy, multicomponent interventions, and mindfulness-based intervention). In particular, social support interventions are important, and they have proven to be effective because carers of people with dementia often rely on their social networks for support [8]. In this regard, peer support interventions seem especially promising and are therefore increasingly being used [9]. However, face-to-face social support interventions are not always feasible because carers may find it difficult to leave the person with dementia alone or under the care of someone else to attend intervention meetings or because services for carers may simply be unavailable or difficult to access, especially in rural regions. Therefore, in recent years, various forms of technology (internet, telephone) have been increasingly used to deliver social support or group interventions. A review by Lee [10] showed that technology-based support group interventions have a positive impact on reducing the care burden among family carers of people with dementia and improving support networks, similar to the way face-to-face support groups connect participants.

Against this background, and because no studies in the field of nursing care have investigated the effects of aftercare following a medical rehabilitation (rehab) measure for carers of people with dementia, in this study, we developed and examined a telephone-based peer support intervention as an aftercare measure (called Talking Time REHAB). Medical rehab specifically designed for family carers of people with dementia is a very new form of medical rehab in Germany (in 2012, this form of care was developed in a rehab facility in the state of Schleswig Holstein). A special feature of this rehab measure is that to facilitate access, the relative with dementia can stay and be cared for in spatial proximity to the family carer. The results of an evaluation study showed that the measure is effective with regard to health-related and psychosocial outcomes but that it is not sustainable. The effects were no longer detectable 6 months after discharge from the rehab facility [11]. The system of medical rehab is an exclusively German procedure, and it is a well-known phenomenon that strong effects are achieved at the end of rehab but then decline, with patients showing regression to a prior disease status, sometimes up to the initial burden [12, 13]. With aftercare, this decline should be avoided, and the rehab effects should be maintained.

Rehab aftercare can support the transfer of what has been learned in rehab into everyday life. Therefore, the goal of the Talking Time REHAB project was to support the transfer of what is learned in rehab to the home environment with the help of location-independent telephone-based aftercare group sessions and thus to perpetuate the rehab effects.

In this paper, we present the results of the effectiveness evaluation.

## Materials and methods

### Study design

**Fig. 1:**
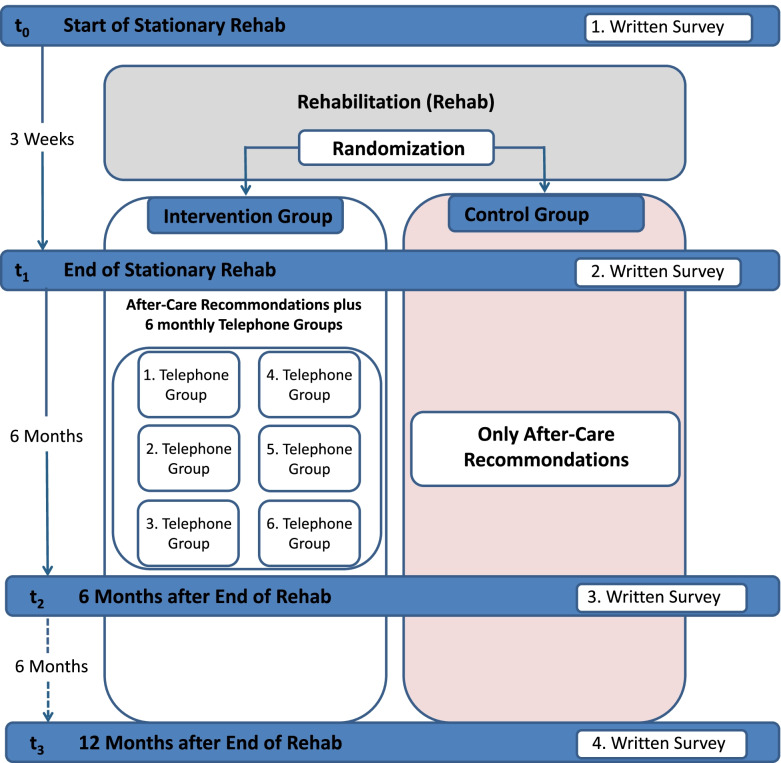
Study design (from Berwig and colleagues [14])

From 2018 to 2021, a prospective longitudinal randomized controlled study with four measurement time points was conducted in a psychosomatic rehab facility for family carers of people with dementia (rehabilitants). Measurements were taken at baseline (t0), at the end of rehab (t1) (mostly approximately 3 weeks after baseline), 6 months after rehab (t2), and 12 months after rehab (t3) (see Fig. 1). Study participants were randomised into two treatment groups (control versus intervention), and the corresponding single random allocation sequence (simple randomisation) was generated with the SPSS software package, version 22.0.

### Participants and recruitment

The inclusion of rehabilitants in the study was consecutive after the provision of informed consent to participate in the study; family carers who underwent the rehab programme in the company of their relative with dementia were included. Only family carers of a person with dementia who had been diagnosed with a mental disorder (usually an adjustment disorder) could participate in the rehab programme. The exclusion criterion was an inability to complete the questionnaires due to personality disorder, psychotic symptoms, language barriers or cognitive impairments.

All newly arriving rehabilitants at the clinic were screened by the study staff on site regarding their eligibility for study participation according to the inclusion/exclusion criteria. Eligible rehabilitants were then scheduled to attend the information session via the therapy control centre. During the information session, the rehab patients were informed about the study project and asked for their consent to participate in the study.

### Study procedures

The intervention was evaluated using a written six-page questionnaire at the four measurement points. The questionnaires for the first (T0) and second time points (t1) were given to all rehabilitants who gave their informed consent to participate in the first information session (see above). The participants then had time over the weekend to complete the t0 questionnaires and return them to the social workers at the clinic or the study staff on site. The t1 questionnaires were returned by the participants at the end of rehab to the study staff, who simultaneously requested their randomized assignment to the intervention group (IG) or the control group (CG) from the Institute for Social Medicine and Epidemiology (ISE) of the University of Lübeck. The randomization result was communicated to the study participants in a further information session. At this session, the participants in the CG were given an information sheet on the further course of the study, after which they left the information session. The participants in the IG were then each given a group folder and a notebook with information material and a space for notes to support their participation in the groups. The notebook contained, among other things, the relevant dates and explanations.

The t2 and t3 questionnaires for the 6-month and 12-month follow-ups were sent to all study participants from the social workers of the rehab facility.

### Intervention

We used the intervention previously described by Berwig and colleagues in the study protocol for this study [14].

### Intervention group

At the end of the rehab stay, the participants of the IG and the CG received a portfolio of aftercare recommendations, such as useful support services, as well as recommendations for self-care in everyday nursing and caregiving. The aftercare recommendations were discussed intensively at the end of the rehab programme. The participants in the IG were also given the dates of the telephone-based aftercare group sessions during this discussion.

The IG participated in six aftercare group sessions, which were conducted as telephone conferences once a month over a six-month period. The after-group sessions were moderated by qualified social workers based on a manual. For the purpose of quality assurance, the moderators were supervised regularly by a consultant psychiatrist after the first and fourth group sessions, and each session was logged by the moderators (immediate and recalled protocol). In this way, adherence to the manual was monitored. The first aftercare group session allowed the group members to get to know each other, to discuss the content of the process, and to exchange information about arriving at home after rehab. Each additional aftercare group session focused on a defined topic: implementation of aftercare recommendations, dealing with the relative with dementia, self-care, dealing with grief and loss in dementia, and social networks. The groups consisted of a maximum of five family carers and the moderator who led the group. The total duration of each aftercare group session was approximately 60 min. At the beginning of each group discussion, each participant gave a brief summary of his or her current care situation and the status of the implementation of his or her aftercare recommendations (approximately 10–20 min) in the initial round. This was followed by a short keynote presentation by the moderator on the respective session topic (approx. 10 min) and then a topic-centred moderated exchange of experiences between the family carers. A total of 21 telephone-based aftercare groups were planned, but only a total of 18 groups could be formed because three groups were merged due to lack of sufficient participants.

### Control group

The family carers of the CG only received a portfolio with aftercare recommendations that were adapted to the individual situation of the respective family carer at the end of rehab. These recommendations were then implemented independently by the family carers and were not linked to an aftercare measure. Between t1 and t2, the family carers in the CG group continued their usual activities at home or continued to use the usual services offered without restriction (usual care).

### Outcomes

Quantitative measurement instruments, previously described by Berwig and colleagues [14], were used to evaluate the intervention effects. The instruments were selected on the basis of their suitability for the scientific question, the intervention carried out, the sample, the data collection procedure, and their psychometric properties. The primary outcome measure was defined as restrictions in social participation [15]. The top rehab tasks in the German rehab system are social participation and self-determination. This is based on the biopsychosocial model of functional health, which is firmly anchored in German social law. The restriction of social participation in particular is a frequent and extremely stressful consequence of assuming care responsibility. The secondary outcome measures included depression [16], health-related quality of life [17], general complaints [18], and performance [19]. All scales used in this study have been found to have good reliability coefficients, with Cronbach’s alphas between 0.83 (IMET) and 0.90 (CESD). In addition to the primary and secondary outcomes, utilization of support services, risk factors and sociodemographic variable services were recorded with individual items.

In the questionnaire for the survey of the primary and secondary outcomes, we also captured satisfaction with the rehab and aftercare concept (T3). In addition, the participants in the IG were asked about their satisfaction with and the feasibility of the aftercare intervention. Furthermore, separately from the outcome evaluation, we carried out a comprehensive process evaluation. For example, after each telephone after-care group session, the participants were asked about their satisfaction with the respective group session using a one-page short questionnaire, and qualitative interviews with the participants of the IG were conducted after the end of the intervention (for more details, please refer to the study protocol of this study [14]). The results of this process evaluation are summarized in two separate manuscripts, which have already been submitted for publication elsewhere.

### Statistical effect analysis

Details of the sample size calculation are included in the published study protocol [14]. Briefly, in terms of differences in standardized response means (SRM), we assumed a between-group difference from baseline to the 6-month follow-up of 0.44 (SRM in the IG: 0.50, SRM in the CG: 0.06). For a two-sided parametric test of group differences at the 5% level, a targeted power of 0.80 and an allocation ratio of 1:1 resulted in a sample size estimate of 2 × 82 study participants. Assuming a drop-out rate of 20% at the 12-month follow-up, we aimed to recruit 103 participants each for the IG and CG.

Analyses were performed using the SPSS software package, version 22.0. Descriptive statistics and correlation analyses were calculated (chi-square test, Student’s t-test, repeated-measures analysis of variance). In addition, intragroup effect sizes were calculated, with mean differences standardized at the pooled standard deviations [20]. Effect sizes were interpreted according to Cohen [21]: d > 0.2, small effect; d > 0.5, medium effect; and d > 0.8, large effect. The significance level was set at *p* < 0.05.

## Results

### Participants and drop-outs

**Fig. 2:**
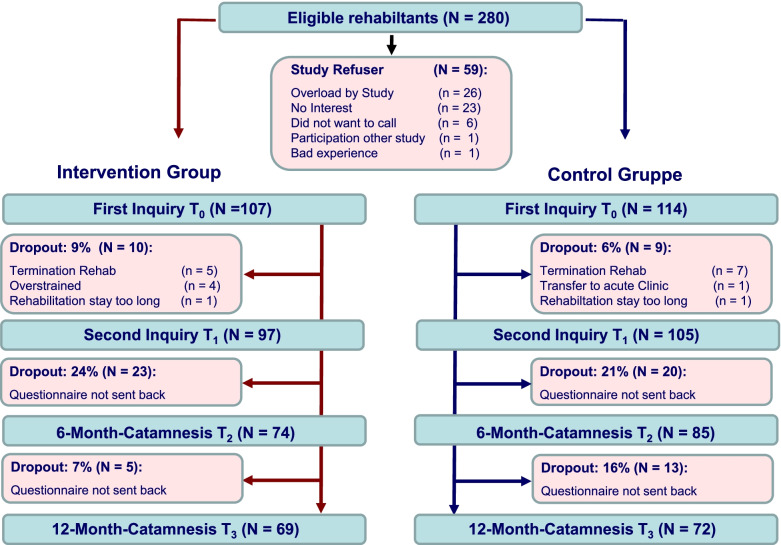
Study flow. Legend: N/n = number, T = time point

Recruitment started in June 2018 and was completed in August 2019. A total of 280 eligible rehabilitants were addressed to participate in the study. Fifty-nine of the 280 rehabilitants did not want to participate in the study. In total, 221 rehabilitants were willing to participate in the study after being informed about the study and data protection, with 107 in the IG and 114 in the CG. Figure 2 shows the flow chart of the study procedure.

At the end of rehab, 19 participants dropped out of the study, 10 from the IG and nine from the CG. Six months after the end of rehab, 43 family carers no longer participated in the study, including 23 from the IG and 20 from the CG. At 12 months after rehab, another 18 participants dropped out of the study because they did not return the follow-up questionnaire. Five of these participants were from the IG, and 13 were from the CG. No statements can be made about the reasons for the dropout at either measurement time point, as the questionnaires were not returned and the participants did not want to be asked about this by the study team. A dropout analysis revealed no significant differences between participants and dropouts in the IG and CG with regard to sociodemographic and health-related characteristics. There were also no differences in sociodemographic and disease-related characteristics at either time point (data not shown).

### Sample characteristics

Three-quarters of the respondents were female, and the average age was 74. Most respondents had a secondary school diploma or intermediate school leaving certificate and had completed vocational school. A minority were currently employed (*N* = 13). The IG and CG did not differ in sociodemographic characteristics, except for income. Table 1 shows the sample characteristics of the rehabilitants included.

**Table 1:** Sample characteristics

	IG *N* = 69	CG *N* = 72	*p*-value^*^
Sex female, N (%)	47 (69.1)	58 (80.6)	0.118
Age, years, M (R)	73.1 (52–85)	74.4 (52–90)	0.304
Marital status, N (%)			
*Married*	59 (86.8)	68 (94.4)	0.290
*Living alone*	5 (7.4)	2 (2.8)	
*Living with a steady partner*	4 (5.9)	2 (2.8)	
Net household income, N (%)			
*500 to < 1500 €*	11 (16.2)	2 (2.8)	0.010
*1500 to < 3000 €*	42 (61.8)	43 (60.6)	
*3000 € or more*	15 (22.1)	26 (36.6)	
School education, N (%)			
*Up to 9 years*	28 (41.8)	23 (31.9)	0.470
*10–11 years*	28 (41.8)	34 (47.2)	
*12–13 years*	11 (16.4)	15 (20.8)	
Vocational training, N (%)^b^			
*None*	6 (9.1)	5 (7.1)	0.865
*Post-secondary non-tertiary*	45 (68.2)	47 (67.1)	
*First stage of secondary*	15 (22.7)	18 (25.7)	
Gainfully employed, N (%)	9 (13.4)	4 (5.6)	0.117

*IG* intervention group, *CG* control group, *N* number

^*^F−/chi-square test, ^b^classification according to ISCED levels from 1997

### Start of rehab

#### Care situation

Most respondents cared for their spouse. The length of time care had been provided to the relative at home averaged 5.5 years, and the range was from less than 1 year to 38 years. Carers spent an average of 14 h on care per day, with 29% of respondents engaging in caregiving around the clock. Regarding the care situation, the CG showed a slightly longer average duration of care (6.3 years vs. 4.6 years, *p* = 0.043), with no differences between the IG and CG in terms of daily hours spent.

Most family carers made use of support offers for the care of their relatives with dementia prior to rehab (see Table 2).

**Table 2:** Support services used before rehab and over time

% Yes	Group	Measurement time points, % Yes			***p***-value^**1**^			***p***-value^**2**^	
		T0	T2	T3	T0	T2	T3	T0-T2	T0-T3
Family, friends, neighbours	IG	60.9	78.3	76.5	0.373	**0.018**	0.181	**< 0.01**	**0.027**
	KG	68.1	59.7	66.2				0.210	1.0
Alzheimer’s Society	IG	11.6	11.6	11.8	0.517	0.199	0.339	1.0	1.0
	KG	8.3	5.6	7.0				0.687	1.0
Alzheimer’s Family Initiative	IG	5.8	11.6	7.4	0.258	0.535	0.811	0.289	1.0
	KG	11.6	8.5	8.5				0.774	1.0
Relatives’ group	IG	24.6	26.1	30.9	0.735	0.821	0.359	1.0	0.344
	KG	22.2	27.8	23.9				0.424	0.815
Nursing advice centre	IG	23.2	21.7	25.0	0.182	0.234	0.240	1.0	1.0
	KG	33.3	30.6	16.9				0.832	**0.036**
Pastoral care	IG	4.3	7.2	7.4	0.614	0.085	0.085	0.500	0.500
	KG	2.8	1.4	1.4				1.0	1.0
Outpatient psychotherapy	IG	7.2	13.0	13.2	0.598	0.412	0.546	0.289	0.289
	KG	9.7	18.1	16.9				0.180	0.267
Support employer	IG	–	2.9	2.9	–	0.146	0.146	–	–
	KG	–	–	–				–	–
Other	IG	5.8	4.3	5.9	0.656	0.614	0.764	1.0	1.0
	KG	4.2	2.8	7.1				1.0	0.687

^1^Chi^2^ between groups, df = 1 (statistically significant values are printed in bold)

^2^McNemar within groups, df = 1 (statistically significant values are printed in bold)

#### Primary and secondary outcomes

With regard to the primary and secondary outcome measures, the starting positions of the IG and CG were comparable at the beginning of the study. Both groups showed clear strain, with the performance at the beginning of rehab being clearly limited, especially with regard to leisure and work. In these two areas, the performance losses reached 50%; in the case of performance in everyday life, the losses were less pronounced, at 30%. There were no statistically significant differences between the two groups at baseline (see Table 3).

**Table 3:** Primary and secondary outcomes over time (additional information on F-values, df and *p*-values can be obtained from Additional file 1: Table S1)

	Group	Measurement time points, means, ***p***-values								SRM			***p***-values^**a**^		
		T0	p	T1	p	T2	p	T3	p	T0-T1	T0-T2	T0-T3	time	group	time*group
Restrictions on Social Participation (IMET)^b^	IG	42.1	.598	**	**	36.7	.613	38.9	.792	**	0.27	0.16	0.051	0.919	0.643
	CG	40.3		**		37.5		39.4		**	0.11	0.05			
General health complaints (SCL)^b^	IG	9.7	.231	6.5	.026	9.0	.783	9.3	.704	0.63	0.14	0.11	< 0.01	0.234	0.259
	CG	7.6		4.8		8.1		9.1		0.72	−0.13	−0.23			
Psychological distress (CES-D)^b^	IG	24.2	.216	13.2	.449	19.8	.122	20.8	.026	1.22	0.44	0.40	< 0.01	0.452	**< 0.01**
	CG	22.1		14.0		22.4		23.6		0.93	−0.06	−0.18			
Physical health (WHOQOL-BREF)^c^	IG	56.1	.203	**	**	61.6	.856	61.5	.926	**	0.37	0.42	< 0.01	0.591	0.079
	CG	60.6		**		61.3		61.6		**	0.04	0.10			
Mental health (WHOQOL-BREF)^c^	IG	56.6	.865	**	**	60.9	.079	62.3	.018	**	0.26	0.41	0.107	0.146	**0.011**
	CG	56.5		**		56.6		55.7		**	0.0	−0.09			
Social relations (WHOQOL-BREF)^c^	IG	51.9	.763	**	**	55.2	.580	57.1	.474	**	0.17	0.34	0.117	0.667	0.226
	CG	53.5		**		52.9		54.1		**	−0.04	0.06			
Environment (WHOQOL-BREF)^c^	IG	68.0	.391	**	**	72.1	.914	71.3	.875	**	0.35	0.27	0.020	0.745	0.250
	CG	70.7		**		71.8		70.9		**	0.10	0.05			
Social support (FSozU)^dc^	IG	80.5	.179	**	**	84.4	.619	85.3	.656	**	0.35	0.41	0.017	0.819	**< 0.01**
	CG	84.1		**		83.7		84.0		**	−0.06	−0.01			
Performance – daily life^c^	IG	6.3	.438	**	**	7.1	.075	6.8	.500	**	0.33	0.23	0.185	0.779	0.126
	CG	6.6		**		6.6		6.7		**	0.0	0.01			
Performance – leisure time^c^	IG	3.9	.680	**	**	5.0	.040	4.7	.329	**	0.46	0.33	0.081	0.626	**0.011**
	CG	4.2		**		4.1		4.2		**	−0.02	0.06			
Performance – occupation^c^	IG	4.9	.170	**	**	7.0	.080	6.6	.169	**	0.76	0.61	0.543	0.965	0.167
	CG	6,3		**		5,3		5,3		**	−1.0	−1.0			

*SRM* standardized response mean, *p* probability, *IMET* Instrument zur Messung der Einschränkungen der Teilhabe [instrument for measurement of limitations of participation], *SCL* symptom checklist, *CES-D* Center for Epidemiologic Studies - Depression Scale, *WHOQOL-BREF* World Health Organization - Quality of Life Scale, *IG* intervention group, *CG* control group

^a^F-tests (statistically significant values are printed in bold)

^b^high values indicate high impairments, ^c^ high values indicate low impairments

^**^not collected at T1

#### Pain

At the beginning of rehab, the participants suffered from clearly pronounced pain, most severely from back, shoulder and neck pain. In general, a high prevalence of pain was observed for all pain localisations recorded (see Table 4). An average of 7.5 pain localisations was mentioned. The IG and CG did not differ in the extent of pain.

**Table 4:** Pain over time

Pain location	Group	Measurement times, mean values, % yes				***p***-value^**a**^				***p***-value^**b**^		
		t0	t1	t2	t3	t0	t1	t2	t3	t0–t1	t0–t2	t0–t3
Face	IG	33.3	17.4	33.3	43.5	0.862	0.472	0.235	0.541	**0.013**	1.0	0.189
	CG	34.7	22.2	43.1	48.6					**0.049**	0.238	0.087
Head	IG	63.8	59.4	53.6	59.4	0.282	0.643	**0.035**	0.109	0.629	0.189	0.664
	CG	72.2	55.6	70.8	72.2					**0.023**	1.0	1.0
Neck	IG	81.2	66.7	78.3	73.9	0.377	0.605	0.648	0.250	**0.013**	0.774	0.267
	CG	75.0	62.5	75.0	81.9					0.064	1.0	0.405
Shoulders	IG	76.8	62.3	78.3	76.8	0.953	0.516	0.519	0.660	**< 0.01**	1.0	1.0
	CG	76.4	56.9	73.6	73.6					**< 0.01**	0.791	0.804
Back	IG	82.6	68.1	82.6	78.3	0.734	0.596	0.734	0.088	**0.021**	1.0	0.581
	CG	84.7	63.9	84.7	88.9					**< 0.01**	1.0	0.508
Chest	IG	42.0	14.5	47.8	42.0	0.772	0.434	0.687	0,149	**< 0.01**	0.481	1.0
	CG	44.4	19,4	44.4	54.2					**< 0.01**	1.0	0.281
Arm	IG	50.7	31,9	53.6	53.6	0.931	0.865	0.285	0.369	**< 0.01**	0.856	0.856
	CG	50.0	30.6	62.5	61.1					**< 0.01**	0.078	0.185
Hands	IG	56.5	44.9	55.1	56.5	0.287	0.661	0.113	0.077	0.096	1.0	1.0
	CG	65.3	48.6	68.1	70.8					0.012	0.804	0.503
Stomach	IG	44.9	20.3	46.4	44.9	0.823	0.634	0.667	0.154	**< 0.01**	1.0	1.0
	CG	43.1	23.6	50.0	56.9					**< 0.01**	0.424	0.087
Abdomen	IG	33.3	20.3	33.3	36.2	1.0	0.312	0.605	0.137	0.078	1.0	0.815
	CG	33.3	13.9	37.5	48.6					**< 0.01**	0.678	**0.043**
Hips	IG	53.6	33.3	52.2	68.0	0.818	0.724	0.367	0.772	**< 0.01**	1.0	0.648
	CG	55.6	30.6	59.7	55.6					**< 0.01**	0.607	1.0
Legs	IG	69.6	56.5	66.7	66.7	0.292	0.779	0.200	0.593	0.064	0.824	0.815
	CG	61.1	54.2	76.4	70.8					0.359	**0.027**	0.189
Feet	IG	63.8	50.7	62.3	62.3	0.745	0.677	0.372	0.590	0.064	1.0	1.0
	CG	61.1	47.2	69.4	66.7					**0.031**	0.263	0.481

*p* probability

^a^Chi-square between groups (statistically significant values are printed in bold), df = 1

^b^McNemar within groups, df = 1 (statistically significant values are printed in bold)

#### Behavioural problems of the relative with dementia

Family carers reported a range of behavioural abnormalities in those with dementia. The most common were forgetfulness, impaired daily living skills and personality changes. With the exception of the tendency to run away, there was no difference between the behavioural problems reported by the IG and CG (see Table 5).

**Table 5:** Behavioural problems of the relative with dementia over time

% Very strong to strong	Group	Measurement time points, % yes				***p***-value^**a**^				***p***-value^**b**^		
		t0	t1	t2	t3	t0	t1	t2	t3	t0–t1	t0–t2	t0–t3
Aggressiveness	IG	17.4	5.9	7.6	16.1	0.939	0.542	0.284	0.746	**0.021**	0.065	0.727
	CG	16.9	8.6	13.2	14.1					0.227	0.754	1.0
Day-night rhythm	IG	22.1	2.9	22.7	24.2	0.254	**0.043**	0.051	0.485	**< 0.01**	1.0	1.0
	CG	30.6	12.1	38.2	19.0					**< 0.01**	0.332	0.092
Forgetfulness	IG	79.7	65.2	78.8	85.5	0.459	0.829	0.431	0.942	**0.012**	1.0	0.581
	CG	84.5	63.4	84.1	85.9					**< 0.01**	1.0	1.0
Personality change	IG	54.4	19.1	59.1	54.2	0.393	0.763	0.838	0.197	**< 0.01**	0.481	0.804
	CG	47.1	17.1	57.4	65.5					**< 0.01**	0.096	**< 0.01**
Tendency to run away	IG	2.9	2.9	6.1	8.3	**0.030**	0.684	0.259	0.431	1.0	0.687	0.453
	CG	12.9	4.1	11.6	12.7					**0.031**	1.0	1.0
Limited everyday competence	IG	32.1	55.9	77.6	73.0	0.958	0.910	0.911	0.759	0.146	**0.022**	0.180
	CG	32.9	54.9	76.8	75.4					0.167	0.077	0.118
Incontinence	IG	33.8	35.8	50.7	60.3	0.858	0.251	0.346	0.277	0.678	**< 0.01**	**< 0.01**
	CG	32.4	26.8	42.6	50.8					0.344	**0.039**	**< 0.01**
Difficulties with feeding	IG	14.7	13.2	22.4	30.2	0.925	0.062	0.609	0.265	0.625	0.388	**< 0.01**
	CG	15.3	4.3	18.8	21.5					**< 0.01**	0.388	**0.039**
Bedriddenness	IG	11.8	5.9	9.1	12.9	0.870	0.966	0.733	0.710	0.289	0.727	1.0
	CG	12.7	5.7	7.5	10.8					0.063	0.289	1.0
Impaired balance	IG	36.2	31.8	38.8	43.5	0.385	0.281	0.118	0.920	0.607	1.0	0.267
	CG	43.5	23.6	52.2	44.4					**< 0.01**	0.167	1.0
Hallucinations	IG	8.8	4.5	13.6	11.5	0.843	0.649	0.886	0.567	0.375	0.508	1.0
	CG	9.9	3.0	14.5	15.0					0.219	0.289	0.070
Risk of falling	IG	31.3	23.9	27.3	37.1	0.964	0.993	**0.028**	0.362	0.125	0.508	0.267
	CG	31.0	23.9	45.6	45.2					0.180	**0.013**	**0.039**
Delusions	IG	8.8	2.2	12.1	17.7	1.0	0.296	0.949	0.455	0.375	0.754	0.063
	CG	8.8	0.7	11.8	12.9					0.125	0.453	0.289
Paranoid experience	IG	14.5	5.9	10.8	16.4	0.779	0.393	0.685	0.745	0.070	0.727	1.0
	CG	12.9	2.9	13.0	14.3					**0.039**	1.0	0.774

*p* probability, *t* time point

^a^Chi-square between groups (statistically significant values are printed in bold), df = 1

^b^McNemar within groups, df = 1 (statistically significant values are printed in bold)

### End of rehab

#### Primary and secondary outcome measures

At the end of rehab, the IG and CG achieved significant improvements, with medium to large effect sizes for all subjective health scales. The strongest change was seen in depressive moods (see Table 3).

#### Pain

With regard to pain, rehab patients in both groups reported very strong improvements in some pain localisations. In particular, back, neck and shoulder pain and leg pain improved (see Table 4). The number of pain localisations mentioned also decreased, with slightly more than five types of pain in both groups at the end of rehab.

#### Behavioural problems of the relative with dementia

At the end of rehab, family carers in both groups reported that the behavioural problems of the relative with dementia were reduced in most areas. Strong improvements could be seen in the disorders of the day-night rhythm, forgetfulness and personality changes (see Table 5).

### Six months after the end of rehab

#### Care situation

Six months after rehab, carers provided an average of 12 h of care per day, 2 h less than before rehab. The proportion of those who were engaged with their loved ones around the clock decreased from 38 to 23%.

Six months after rehab, almost a quarter of carers stated that they had not made use of any support offers for themselves. As before rehab, family, friends and neighbours played the most important role and had increased in importance for the IG and decreased in the CG. This difference reached statistical significance (see Table 2).

#### Primary and secondary outcomes

Six months after rehab, the IG and CG descriptively show different developments. In the primary outcome, restrictions on social participation, the intervention yielded a small-sized reduction in restrictions on social participation from baseline to 6-month follow-up (SRM = 0.27), whereas the reduction from baseline to 6-month follow-up in the CG was negligible (SRM = 0.16). For all secondary outcome measures, the IG descriptively achieved an improvement of small magnitude in most cases, while the CG returned to pre-rehab levels or even deteriorated beyond them. The changes over time between the groups did not reach statistical significance in most cases, with the exception of psychological distress, mental health, perceived social support and performance in leisure time (see Table 3).

#### Pain

Six months after rehab, pain had increased significantly. In some cases, it reached the same level as before rehab, and in others, it even exceeded it. A significant difference between the IG and CG at the third measurement point was shown for headaches (see Table 4). A total of eight pain localisations were mentioned. The IG and CG did not differ in the extent of pain.

#### Behavioural problems of the relative with dementia

Six months after rehab, the behavioural problems of the relative with dementia increased again significantly, in some cases exceeding the initial value. Particularly conspicuous and statistically significant were the deterioration with regard to daily living skills and a higher degree of incontinence. These deteriorations affected the relatives from the IG and CG equally (see Table 5). The participants in the IG reported a significantly lower risk of the person with dementia falling than those in the CG at T2.

### Twelve months after the end of rehab

#### Care situation

Even 12 months after rehab, family carers provided an average of 12 h of care per day, 2 h less than before rehab. The proportion of those engaged with their relatives around the clock increased again slightly compared with that at the 6-month follow-up but was still significantly lower than the amount reported before rehab.

Twelve months after rehab, 20% of family carers did not make use of any support services themselves. In addition to the family network, relatives’ groups and care counselling centres played a significant role at this point, with a 30% utilization rate (see Table 2).

#### Primary and secondary outcome measures

Twelve months after rehab, different developments were found for the IG and CG. In the primary outcome, social participation, the IG achieved an improvement at the second follow-up, with a small effect size, whereas the CG continued to deteriorate and almost reached the baseline value before rehab. For all secondary outcome measures, the IG achieved an improvement of a small magnitude in most cases, with medium effect sizes for quality of life, social support, and job performance, while the CG again reached baseline or even continued to deteriorate beyond it. As mentioned above, the changes over time between the groups reached statistical significance for four secondary outcomes (see Table 3).

#### Pain

Twelve months after rehab, the picture was similar to that at the 6-month follow-up. The pain prevalence was similar to the baseline value or slightly exceeded it. The IG and CG did not significantly differ at T3 with regard to pain. Table 4 shows the changes over time.

Eight types of pain were mentioned by both groups at the 12-month follow-up.

#### Behavioural problems of the relative with dementia

Twelve months after rehab, the behavioural problems of the relative with dementia continued to increase in most areas and, in many cases, worsened beyond baseline. Particularly striking and statistically significant were deteriorations with respect to, higher levels of incontinence, and difficulty with feeding. These deteriorations affected the IG and CG relatives equally. The IG and CG did not significantly differ at T3 with regard behavioural problems of the relative with dementia in the respective sections. Table 5 summarises the changes.

#### Satisfaction with the rehab and aftercare concept

Engagement in rehab together with the relative with dementia was rated very positively by most family carers. Most participants from the IG and CG stated that they would not have used rehab without their relative with dementia accompanying them. Almost 80% were able to take advantage of the treatments in a relaxed manner because they knew that their relative was well cared for. In the assessment of joint therapy offers together with the person with dementia, there was a significant difference between the IG and CG. While almost 70% of the IG found the joint offers helpful, only just under 50% of the CG did (*p* = 0.024).

Aftercare in the form of telephone-based aftercare groups was evaluated very positively by the participants of the IG. These groups seemed to be a suitable and useful method for integrating what has been learned in rehab into everyday care and to provide family carers with support and safety. The choice of the telephone as a medium is optimal for this target group, as they are usually older. The topics defined together with the rehabilitants are precisely tailored to the needs and problems of the family carers.

## Discussion

As a result of the telephone aftercare group sessions, a lasting effect (effect size ES = 0.50) was expected for the primary outcome, social participation, in the IG in comparison to the CG. This assumption could not be confirmed: There was a slightly nonsignificant time effect but no interaction effect. The progression of dementia, as a defining symptom of dementia syndrome [22], can be used as an explanation for this finding. That is, the care demands on family carers increase as a result of the rapid progression of the disease [23], thereby reducing the effect of the intervention. Furthermore, aftercare recommendations from the rehab with regard to the use of support and respite services could not be implemented to the required extent. Since increased social participation is associated with the relief of nursing and care tasks, this could explain the lack of an intergroup intervention effect on the primary outcome and the existing but small-sized reduction in restrictions on social participation in the IG.. We also assume that insufficient offerings of support and respite services on site [24] are responsible for the unchanged low utilization of support and relief services. This is especially true if family carers live in rural areas.

While social participation could be increased by telephone-based aftercare group sessions, but not to the expected extent, clear advantages for the IG can be shown in the secondary outcomes. For almost all health-related outcomes captured, the IG achieved medium or at least small effects, while the CG no longer showed any effects or even deteriorated. Pain also has to be evaluated against the background of worsening dementia. Both groups showed a significant improvement in almost all pain areas immediately at the end of the rehab, but pain reached the initial values again after 6 and 12 months or continued to deteriorate. It can be assumed that the frequency of occurrence of problem behaviours and the care effort of the relative with dementia contribute to this. The positive developments directly at the end of the stationary rehab stay show the importance of care relief. If this does not succeed to a sufficient degree at home, there is no longer any possibility of adequate self-care. As a result, pain and health problems increase again because stress and pain reactions overlap substantially [25].

As can be expected in a randomized study, the IG and CG did not differ in almost any sociodemographic characteristics. Only net household income was slightly higher in the CG. This difference reached statistical significance (see Table 1). Because income was not associated with any of the outcome variables, it is unlikely that our general results are confounded by this difference.

Overall, the effect of telephone-based aftercare group sessions, as a peer support intervention, seems to be mainly reflected in the improvement of general health-related outcomes. These results point in the same direction as those of a feasibility study by the senior author of this study, in which telephone support groups for family carers of people with dementia in the format of Talking Time REHAB were tested [26]. A review on peer support interventions also suggests this [9]. In a peer support intervention, the psychoeducational aspect of the intervention is less important than the social support provided by peers or other family carers, who are in a similarly challenging situation. This may have an effect on the concrete and increased experience of social support in the group as well as the experience of the universality of suffering [27]. This is a central effective factor of group psychotherapy and means that participants in the telephone-based aftercare group sessions experience relief in that other participants are confronted with similar problems, others have similar feelings, and they are not the only ones suffering, as others also have problems and crises in caring for their relative with dementia.

In relation to existing aftercare measures (e.g., telephone aftercare counselling, on-site outpatient aftercare programmes), Talking Time REHAB is innovative as a telephone-based aftercare measure in a group format. To our knowledge, such a form of aftercare in a group format or as a peer support intervention has not existed before. We believe that our results regarding this form of group aftercare are promising and show that this type of aftercare could also be helpful for relatives caring for people with other diseases (e.g., family carers of stroke survivors, people with other neurodegenerative diseases or cancer). In this respect, the present study results represent an important contribution to the development of rehab aftercare measures in general. Nevertheless, it makes sense to think about further optimization of the programme. For example, it could be useful to homogenize the aftercare groups, especially with regard to occupation or the type of relationship to the relative with dementia. This is because the problems of family carers who are still employed or care for a relative, e.g., with the behavioural variant of frontotemporal dementia (bvFTD), differ very much from family carers of people with Alzheimer’s or already retired family carers [28, 29]. Accordingly, in these different care situations, the content of telephone-based aftercare would have to be adapted. Further developments, for example, in the form of digital applications, would also be worth considering.

### Strengths and limitations of the study

Several strengths of this study may be emphasized. The sample size was relatively large, the intervention was standardized by means of a manual, the intervention fidelity was controlled by session protocols and continuous supervision, the outcomes were assessed by written surveys of the family carer (e.g., assessments are not prone to interviewer biases) and the data quality was ensured by monitoring by the University of Lübeck, who conducted scientific supervision of the study. Another strength is that an important concept that has not yet been adequately evaluated was being investigated in this study: medical rehab for the family carers themselves in which the relative with dementia could be brought along. Existing rehab measures focus on the relative with dementia himself or herself, not the carer. In this respect and regarding the telephone-based group format of the aftercare measure, the study has a highly innovative character.

One limitation of this study is that parts of the follow-up surveys took place during the coronavirus pandemic. The extent to which this had an impact on individual participants in the study cannot be assessed conclusively on the basis of the available data.

## Supplementary Information

**Additional file 1: Table S1.** Additional statistics to Table 3.

## Data Availability

Not applicable.

## Abbreviations

bvFTD: Behavioural variant of frontotemporal dementia
CG: Control group
ES: Effect size
IG: Intervention group
IMET: Index zur Messung von Einschränkungen der Teilhabe (Measurement of Restrictions on Participation)
ISE: Institute for Social Medicine and Epidemiology
N: Number
p: Probability
Rehab: Rehabilitation
SPSS: Statistical Package for the Social Sciences
t: Time
