# Supplementary material for: Telephone-based aftercare groups for family carers of people with dementia – results of the effect evaluation of a randomised controlled trial
Source: BMC Health Serv Res. 2022 Feb 11;22:177. doi: 10.1186/s12913-022-07490-9 (PMC8831098; doi:10.1186/s12913-022-07490-9)
Supplement: Supplementary file 1 — Additional file 1: Table S1. Additional statistics to Table 3. [file 12913_2022_7490_MOESM1_ESM.docx]

Additional file 1: Table S1. Additional statistics to Table 3

| **Variables** | **Effects** | | | | | | | | |
| --- | --- | --- | --- | --- | --- | --- | --- | --- | --- |
|  | **Time** | | | **Group** | | | **Time*group** | | |
|  | **F** | **df** | **p** | **F** | **df** | **p** | **F** | **df** | **p** |
| Restrictions on Social Participation (IMET) | 3.04 | 2.127 | 0.051 | 0.010 | 1.128 | 0.919 | 0.44 | 2.127 | 0.643 |
| General health complaints (SCL) | 24.57 | 3.97 | <0.01 | 1.43 | 1.99 | 0.234 | 1.36 | 3.97 | 0.259 |
| Psychological distress (CES-D) | 64.66 | 3.121 | <0.01 | 0.57 | 1.123 | 0.452 | 4.02 | 3.121 | <0.01 |
| Physical health (WHOQOL-BREF) | 4.95 | 2.131 | <0.01 | 0.29 | 1.131 | 0.591 | 2.59 | 2.132 | 0.079 |
| Mental health (WHOQOL-BREF) | 2.27 | 2.131 | 0.107 | 2.13 | 1.132 | 0.146 | 4.67 | 2.131 | 0.011 |
| Social relations (WHOQOL-BREF) | 2.19 | 2.130 | 0.117 | 0.19 | 1.131 | 0.667 | 1.51 | 2.130 | 0.226 |
| Environment (WHOQOL-BREF) | 4.02 | 2.132 | 0.020 | 0.75 | 1.133 | 0.745 | 1.4 | 2.132 | 0.250 |
| Social support (FSozU) | 4.18 | 2.130 | 0.017 | 0.05 | 1.131 | 0.819 | 4.95 | 2.130 | <0.01 |
| Performance – daily life | 1.71 | 2.114 | 0.185 | 0.08 | 1.115 | 0.779 | 2.11 | 2.114 | 0.126 |
| Performance – leisure time | 2.57 | 2.112 | 0.081 | 0.24 | 1.113 | 0.626 | 4.72 | 2.112 | 0.011 |
| Performance – occupation | 0.69 | 2.5 | 0.543 | 0.002 | 1.6 | 0.965 | 2.62 | 2.5 | 0.167 |

F = value of F-distribution; df = degree of freedom; p = probability; IMET = Instrument zur Messung der Einschränkungen der Teilhabe [instrument for measurement of limitations of participation]; SCL = Symptom checklist; CES-D = Center for Epidemiologic Studies - Depression Scale; WHOQOL-BREF = World Health Organisation - Quality of Life Scale
